# Genomic Profiling and Prognostic Value Analysis of Genetic Alterations in Chinese Resected Lung Cancer With Invasive Mucinous Adenocarcinoma

**DOI:** 10.3389/fonc.2020.603671

**Published:** 2021-01-11

**Authors:** Lei Cai, Jiangfeng Wang, Junrong Yan, Jian Zeng, Liang Zhu, Jinxiao Liang, Chao Pan, Xiancong Huang, Ju Jin, Yang Xu, Fufeng Wang, Yang Shao, Qinqin Xu, Guojie Xia, Minyan Xing, Xiaoling Xu, Youhua Jiang

**Affiliations:** ^1^ Department of Thoracic Surgery, Cancer Hospital of the University of Chinese Academy of Sciences (Zhejiang Cancer Hospital), Hangzhou, China; ^2^ Institute of Cancer and Basic Medicine (IBMC), Chinese Academy of Sciences, Hangzhou, China; ^3^ Medical Department, Nanjing Geneseeq Technology Inc., Nanjing, China; ^4^ Department of Pathology, Cancer Hospital of the University of Chinese Academy of Sciences (Zhejiang Cancer Hospital), Hangzhou, China; ^5^ Department of Medical Oncology, Traditional Chinese Medical Hospital of Huzhou, Huzhou, China; ^6^ Department of Medical Oncology, Haining People’s Hospital, Haining Branch, The First Affiliated Hospital, Zhejiang University, Haining, China

**Keywords:** invasive mucinous adenocarcinoma, surgical resection, next generation sequencing, genomic profiling, prognostic biomarker

## Abstract

**Background:**

Invasive mucinous adenocarcinoma (IMA) of the lung is a distinct histological subtype with unique clinical and pathological features. Despite previous genomic studies on lung IMA, the genetic characteristics and the prognosis-related biomarkers in Chinese surgically resected lung IMA remain unclear.

**Methods:**

We collected 76 surgically resected primary tumors of invasive lung adenocarcinoma, including 51 IMA and 25 non-mucinous adenocarcinomas (non-IMA). IMA was further divided into pure-IMA (mucinous features≥90%) and mixed-IMA subgroups. Comprehensive genomic profiling based on targeted next-generation sequencing (NGS) of 425 genes was explored and genomic characteristics were evaluated for the correlation with postoperative disease-free survival (DFS).

**Results:**

IMA had a unique genetic profile, with more diverse driver mutations and more tumor drivers/suppressors co-occurrence than that of non-IMA. The frequency of *EGFR* (72.0% vs. 40.0% vs. 23.1%, p=0.002) and *ALK* (undetected vs. 20.0% vs. 26.9%, p=0.015) alterations showed a trend of gradual decrease and increase from non-IMA to mixed-IMA to pure-IMA, respectively. The frequency of *KRAS* mutations in pure-IMA was higher than that in mixed-IMA, albeit statistically insignificant (23.1% vs. 4.0%, p=0.10). *TP53* mutation was significantly less in pure-IMA compared to mixed-IMA and non-IMA (23.1% vs. 52.0% vs. 56.0%, p=0.03). Besides, IMA exhibited less arm-level amplifications (p=0.04) and more arm-level deletions (p=0.004) than non-IMA, and the frequency of amplification and deletion also showed a trend of gradual decrease and increase from non-IMA to mixed-IMA to pure-IMA, respectively. Furthermore, prognosis analysis in stage III IMA patients showed that patients harboring alterations in *EGFR* (mDFS=30.3 vs. 16.0 months, HR=0.19, P=0.027) and PI3K pathway (mDFS=36.0 vs. 16.0 months, HR=0.12, P=0.023) achieved prolonged DFS, while patients with poorly differentiated tumors (mDFS=14.1 vs. 28.0 months, HR=3.75, p=0.037) or with *KRAS* mutations (mDFS=13.0 vs. 20.0 months, HR=6.95, p=0.027) had shorter DFS. Multivariate analysis showed that *KRAS* mutations, PI3K pathway alterations, and tumor differentiation status were independent factors that have statistically significant influences on clinical outcomes of IMA patients.

**Conclusion:**

Our study provided genomic insights into Chinese surgically resected lung IMA. We also identified several genomic features that may serve as potential biomarkers on postoperative recurrence in IMA patients with stage III disease.

## Introduction

Lung cancer is the leading cause of cancer-related mortality among various malignancies worldwide (1). In addition to surgery and chemoradiotherapy, targeted therapy has now become an effective treatment for lung cancer (2–5), which in turn emphasizes the importance of understanding tumors at gene levels.

According to the lung adenocarcinoma classification system proposed by the International Association for the Study of Lung Cancer (IASLC)/American Thoracic Society (ATS)/European Respiratory Society (ERS) in 2011 (6), invasive mucinous adenocarcinoma (IMA) is considered to be a distinct subtype of lung adenocarcinoma. IMA appears as translucent and grayish jelly-like lesions at gross observation, while the tumor cells are typified by a tall columnar or goblet cell morphology with abundant intracellular or extracellular mucus under the microscope (7). IMA has a low incidence, accounting for only 2%–5% of lung invasive adenocarcinoma, and IMA is associated with poor survival outcomes (6, 8). It has been shown that more than 70% of IMA tumors were associated with the spread-through-air-space pattern, which a poor prognostic predictor for various lung adenocarcinoma subtypes (9). In addition, some distinct clinic-pathological features were observed within the IMA subtype. Beck *et al*. reported spontaneous regression of airspace opacities in some patients with IMA, who tended to have worse overall survival than other IMA patients (10). Several pathological parameters of the MIA tumors, such as the mucin spread size, tumor cell spread size, and invasive size, were also negatively correlated with poor prognosis (11). Therefore, MIA is a clinically important and complex lung adenocarcinoma subtype that needs to be carefully investigated.

A number of studies have suggested that IMA differs from other adenocarcinomas in clinical manifestations and pathology (6, 8, 12, 13). Some studies indicated that certain genetic differences have been observed between IMA and other non-mucinous adenocarcinomas (non-IMA) particularly for driver genes. Early studies found that the mutation frequency of *EGFR* was lower than other adenocarcinomas (undetectable to ~28% in IMA vs. 47%–78% in non-IMA patients) (14, 15), while the frequency of *KRAS* mutations was higherin IMA than other adenocarcinomas (14%–86% in IMA vs. 1.5%–17% in non-IMA patients) (16, 17). These results implied that the mucinous differentiation might be partially relied on *KRAS* aberrations, whereas *EGFR* mutations tended to be involved in driving the development of other adenocarcinoma subtypes. However, these studies only detected a narrow range of genes and did not include other key oncogenic drivers, such as *ALK* fusions. In 2014, high rates of *ALK* fusions and *KRAS* mutations were observed in IMA from a study containing 13 samples, and a rare *CD74-NRG1* fusion was also detected (18). In the same year, a study conducted on 44 pulmonary mucinous adenocarcinomas found that IMA had a lower frequency of *EGFR* mutations and a higher frequency of several other genetic alterations (e.g., *KRAS* mutations, *HER2* mutations, and *ALK* fusions) than other invasive adenocarcinomas (19). These studies revealed that IMA is likely to have a unique genetic profile, especially for the driver genes. Although more and more genetic changes have been detected in IMA, the overall genetic spectrum is still unclear. In 2015, a study of 72 IMA patients from the USA and South Korea showed that *KRAS* mutations were the most common genetic aberrations (63%), whereas in *KRAS*-negative patients several rare gene fusions and mutations were observed, including *CD74*-*NRG1*, *VAMP2*-*NRG1*, *TRIM4*-*BRAF*, *TPM3*-*NTRK1*, and *EML4*-*ALK* fusions, as well as *ERBB2*, *BRAF*, and *PIK3CA* mutations (12). Among those 72 IMA patients, only 2 of them harbored *TP53* mutations, which was much less than that observed in unselected lung adenocarcinomas (12). This study also employed next-generation sequencing (NGS) for gene detection, which increased the coverage and detecting sensitivity compared to previous studies. In 2016, another NGS-based study using a panel of 50 genes identified *KRAS* and *TP53* as the most commonly mutated genes in IMA, as well as several rare *EGFR* gene mutations (20). Of note, this study categorized IMA into pure-IMA (≥ 90% invasive mucinous pattern) and mixed-IMA (> 10% of the non-mucinous invasive component) (20). Specifically, *ALK* rearrangements were found in two of mixed-IMA patients but none in pure-IMA patients, and patients with mixed-IMA had poorer prognosis (20), indicating that IMA patients with different proportions of mucinous components may have different molecular profiles and clinical prognosis, which is worthy of further investigations.

A review of existing studies suggested that the frequency of *EGFR* mutations was lower and the frequency of *ALK* fusions and *KRAS* mutations was markedly higher in IMA patients compared to the general lung invasive adenocarcinomas, whereas the frequency of rare mutations remains controversial. In addition, most of the previous studies were conducted on the Westerner population using small panel molecular assays, while the extensive genetic analysis in the Chinese population is still lacking. In this study, we retrospectively analyzed 76 surgically resected primary tumors from Chinese patients with invasive lung adenocarcinoma, including 51 IMA and 25 non-IMA. Furthermore, based on the presence of a non-mucinous component, IMA was further divided into pure-IMA and mixed-IMA subgroups. Targeted NGS of 425 cancer-relevant genes was performed on these tumors, and their genomic profile was comprehensively investigated and compared among 3 different histological subgroups. We also explored the association between molecular aberrations and postoperative disease-free survival (DFS) in stage III IMA patients. This study could enhance our understanding of the molecular characteristics and the potential prognostic biomarkers of East-Asian IMA patients.

## Methods

### Patients

A total of 79 surgically resected primary invasive lung adenocarcinomas diagnosed between October 2010 to July 2019 at the Cancer Hospital of the University of Chinese Academy of Sciences (Zhejiang Cancer Hospital). All cases were retrospectively collected and were centrally reviewed by two pathologists according to the 2015 WHO classification of lung adenocarcinoma. Specifically, during the screening process, patients with mucinous component higher than 10% were enrolled in the IMA group. Among the 79 patients, three did not pass the quality control of the sequencing analysis, resulting in 51 IMA and 25 non-mucinous adenocarcinomas (non-IMA). In addition, given that some IMAs have certain levels of the non-mucinous component to some extent, IMA can be further classified based on the percentage of mucinous pattern, that is, pure mucinous (≥90% of the invasive mucinous pattern) and mixed mucinous/non-mucinous (>10% of the non-mucinous invasive component). All patients provided informed consent forms in accordance with institutional regulations and study protocols were approved by the Ethical Review Community of Zhejiang Cancer Hospital.

### DNA Extraction and Library Construction

NGS was performed in a centralized testing center (Nanjing Geneseeq Technology Inc.). DNA extraction, library preparation, and targeted-capture enrichment were performed as previously described (21). Briefly, genomic DNA from the white blood cells (WBCs) was extracted using the DNeasy Blood & Tissue Kit (Qiagen) and used as the normal control to distinguish germline mutations. Formalin-fixed and paraffin-embedded (FFPE) samples were de-paraffinized with xylene, and genomic DNA was extracted using the QIAamp DNA FFPE Tissue Kit (Qiagen). DNA was quantified by Qubit 3.0 using the dsDNA HS Assay Kit (Life Technologies), and the quality was evaluated by a Nanodrop 2000 (Thermo Fisher).

Libraries were prepared by the KAPA Hyper Prep kit (KAPA Biosystems) as previously described (22). Briefly, genomic DNA was sheared into fragments using a Covaris M220 instrument. End repair, A-tailing, and adaptor ligation of fragmented DNA were performed using the KAPA Hyper DNA Library Prep kit (Roche Diagnostics). DNA Libraries were then amplified by polymerase chain reaction (PCR) and purified using AgencourtAMPure XP beads.

Customized xGen lockdown probes panel (containing 425 predefined cancer-related genes) were used for selective enrichment. The capture reaction was performed with Dynabeads M-270 (Life Technologies) and the xGen Lockdown Hybridization and Wash kit (Integrated DNA Technologies). Captured libraries were PCR-amplified with KAPA HiFi HotStart ReadyMix (KAPA Biosystems). The purified library was quantified using the KAPA Library Quantification kit (KAPA Biosystems).

### Sequencing and Bioinformatics Analysis

Target enriched libraries were sequenced on the HiSeq4000 platform (Illumina). Sequencing data were demultiplexed by bcl2fastq (v2.19), analyzed by Trimmomatic (23) to remove low-quality (quality<15) or N bases. Then the data were aligned to the hg19 reference human genome with the Burrows-Wheeler Aligner (bwa-mem) (24) and further processed using the Picard suite (available at https://broadinstitute.github.io/picard/) and the Genome Analysis Toolkit (GATK) (25) SNPs and indels were called by VarScan2 (26) and HaplotypeCaller/UnifiedGenotyper in GATK, with the mutant allele frequency (MAF) cutoff as 0.5%. Common variants were removed using dbSNP and the 1000 Genome project databases. Germline mutations were filtered out by comparing to patient’s WBCs controls.

Gene fusions were identified by FACTERA (27) and copy number variations (CNVs) were analyzed with ADTEx (28). The log2 ratio cut-off for copy number gain was defined as 2.0 for tissue samples. A log2 ratio cut-off of 0.6 was used for copy number loss detection. Allele-specific somatic CNVs (SCNVs) were analyzed by FACETS (29) with a 0.2 drift cut-off for unstable joint segments. In pathway analysis, sets of genetic aberrations were included according to the ten previously defined canonical oncogenic signaling pathways (30).

### Statistical Analysis

Quantitative data were presented as median (range) or the number of patients (percentage). Comparisons of proportion between groups were performed using Fisher’s exact test. Survival analysis was performed using Kaplan-Meier curves, and the p-value was determined with the log-rank test, and hazard ratios (HRs) were calculated by Cox proportional hazards model. A two-sided p-value of less than 0.05 was considered significant for all tests unless indicated otherwise. Univariate and multivariate analyses were used to study the associations between different variables and DFS, and the results were presented as HRs and their 95% confidence intervals (CIs). All analyses were performed with R 3.4.0.

## Results

### Patient Characteristics

Baseline demographics and clinical characteristics of 51 IMA and 25 non-IMA patients were summarized in **Table 1**. There were no significant differences between patients with IMA and those with non-IMA with respect to age, sex, stage, smoking status, intravascular tumor thrombus, perineural invasion, differentiation grading, and pleural invasion. The median age of IMA and non-IMA was 61 and 62 years, respectively. Twenty-eight females (55%) were enrolled in IMA cohort and 11 females (44%) were enrolled in non-IMA cohort. More than half of both IMA and non-IMA patients did not have a smoking history. The proportion of stage III patients in IMA cohort seemed more than that in non-IMA cohort, but no statistical significance was observed (p=0.11). The majority of the patients did not have intravascular tumor thrombus or perineural invasion, and slightly more patients had moderately differentiated tumors than those with poorly differentiated tumors. Nearly half of the IMA patients had pleural invasion, whereas there were a lower proportion of the non-IMA patients who had pleural invasion, although the difference was not statistically significant. According to the proportion of mucinous components, patients with IMA were divided into sub-groups of pure-IMA (n=25) and mixed-IMA (n=26).

**Table 1:** Baseline demographics and clinical characteristics of non-IMA and IMA patients enrolled in this study.

Histology	Non-IMA (n=25)	IMA (n=51)	P value
Median age (range)	62 (50–83)	61 (25–79)	0.29
Gender			0.47
Male	14 (56.0%)	23 (45.1%)	
Female	11 (44.0%)	28 (54.9%)	
Clinical stage			0.31
I	14 (56.0%)	24 (47.1%%)	
II	5 (20.0%)	6 (11.8%)	
IIIA	6 (24.0%)	21 (41.2%)	
Smoking status			0.62
Never	14 (56.0%)	33 (64.7%)	
Ever	11 (44.0%)	18 (35.3%)	
Intravascular tumor thrombus			0.37
Yes	3 (12.0%)	11 (21.6%)	
No	22 (88.0%)	40 (78.4%)	
Perineural invasion			0.66
Yes	1 (4.0%)	5 (9.8%)	
No	24 (96.0%)	46 (90.2%)	
Differentiation grading			0.81
Poor	10 (40.0%)	22 (43.1%)	
Moderate	15 (60.0%)	29 (56.9%)	
Well	0 (0.0%)	0 (0.0%)	
Pleural invasion			0.051
Yes	7 (28.0%)	27 (52.9%)	
No	18 (72.0%)	24 (47.1%)	

IMA, Invasive mucinous adenocarcinoma.

### Different Mutational Spectra of Driver Genes Between IMA and Non-IMA

**Figure 1:**
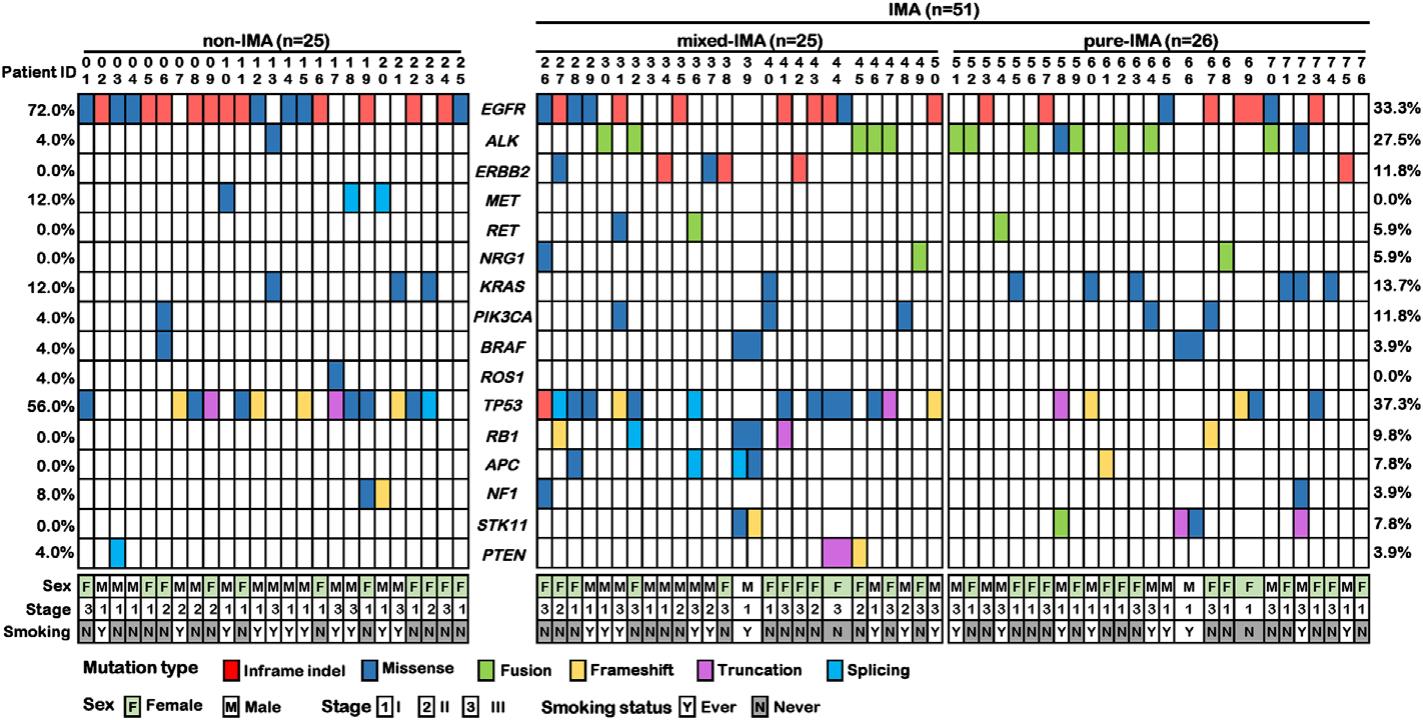
Co-mutation plot of common driver and suppressor genes in invasive mucinous adenocarcinoma (IMA) and non-IMA patients in our cohort. The mutation profile of 25 non-IMA patients and 51 IMA patients were shown. The IMA group could be further divided into mix-IMA (n=25) and pure IMA (n=26) based on the mucinous content of the tumor. Different colors indicate different mutational types. Clinical information on sex, TNM stage, and smoking history was shown at the bottom.

Given that each tumor sample had the corresponding white blood cell sample as the normal control, we used these normal control samples to filter out the germline mutations, and all of our molecular analyses were based on tumor somatic alterations. In IMA patients, the most frequently altered driver genes were *EGFR* (33.3%), *ALK* (27.5%), *KRAS* (13.7%), *ERBB2* (11.8%), and *PIK3CA* (11.8%) (**Figure 1** and **Figure S1**). Among these alterations, the well-known *EGFR* driver mutations (i.e., L858R, exon 19 deletions, and G719S) and *ALK* rearrangements accounted for 31.4% and 23.5%, respectively (**Figure S2**). Specifically, *EGFR* G719S, L858R, and exon 19 deletion (E19 del) were detected in one (2.0%), 3 (5.9%), and 12 (23.5%) patients, respectively. In addition to these known driver mutations, *EGFR* A750P and E19 del coexisted in one patient, and a concomitant *EGFR* L747S mutation was also observed in a patient with *EGFR* G719S. Besides, an *EGFR* exon 20 insertion (20Ins) was detected in 1 (2.0%) patient. In terms of the *ALK* rearrangement, 10 (19.6%) patients harbored *EML4*-*ALK* fusions, one (2.0%) had an intergenic region (IGR)-*ALK* fusion (i.e., downstream *TTC32*-*ALK* exon 18 fusion), and one patient (2.0%) had IGR (downstream *CTNNA2*)-*ALK* exon 20 and *ALK* exon 18-*LYN* 5’ UTR co-fusions. *ERBB2* 20Ins were detected in four (7.8%) patients, which was much higher than that in unselected lung adenocarcinoma patients. In addition, the frequencies of *RET* and *NRG1* alterations were both 5.9%, which includes two patients with *RET* fusions (*KIF5B*-*RET* and *CCDC6*-*RET*) and two patients with *NRG1* fusions (*SLC3A2*-*NRG1*) (**Figure 1** and **Figure S2**). Although *MET* aberrations were not detected in IMA patients, *MET* (12.0%), together with *EGFR* (72.0%) and *KRAS* (12.0%), was commonly mutated in non-IMA patients. The mutation frequency of *ALK*, *PIK3CA*, *BRAF*, and *ROS1* was all 4.0% (**Figure 1** and **Figure S2**), and *MET* exon 14 skipping was detected in two (8.0%) patients (**Figure 1** and **Figure S2**). The frequency of *EGFR* (33.3% vs. 72.0%, p<0.01) and *MET* (undetected vs. 12.0%, p=0.03) mutations in IMA were significantly lower than that in non-IMA patients while the frequency of *ALK* rearrangements showed the opposite trend (27.5% vs. 4.0%, p=0.01). IMA group tended to have more *ERBB2* mutations than non-IMA group, albeit no statistical difference (11.8% vs. 0%, p=0.17). Differences in frequencies of other driver genes were observed, although the results were not statistically significant (**Figure 1** and **Figure S2**). In addition, all the gene mutations detected in three or more patients were shown in **Figure S1**. Among these genes, the mutation frequency of *FBXW7* was significantly lower in IMA than in non-IMA (undetected vs. 12.0%, p=0.03) (**Figure S1**). In terms of gene-level copy number variations, there was no significant difference between non-IMA and IMA in our patient cohort (Data not shown).

### Frequency of Driver Mutations Related to the Proportion of Mucinous Component

**Figure 2:**
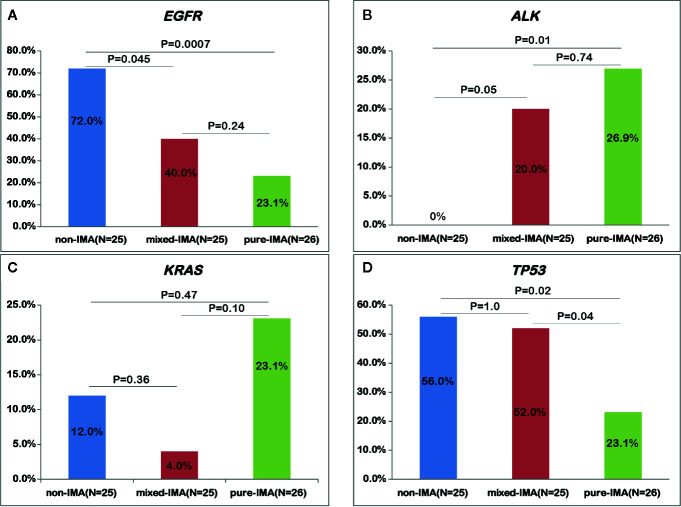
Comparing the alterations of common driver and suppressor genes among samples with different mucinous contents. The mutational frequency of *EGFR* mutation **(A)**, *ALK* fusion **(B)**, *KRAS* mutation **(C)**, and *TP53* mutation **(D)** was compared in patients with non-IMA, mixed-IMA and pure-IMA.

Based on the presence of the non-mucinous component, IMA was further divided into pure-IMA and mixed-IMA subgroups, and comparative analysis of their driver genes was performed. Intriguingly, as the content of the mucinous component increased, the frequency of *EGFR* mutations gradually decreased, with significantly higher frequency in non-IMA comparing with mixed-IMA (72.0% vs. 40.0%, p=0.045) or pure-IMA (72.0% vs. 26.9%, p<0.001). The *EGFR* mutation frequency in mixed-IMA was higher than pure-IMA, but no significant difference was observed (p=0.24) (**Figure 2A**, **Figure S2B**). By contrast, as mucinous content increased, the frequency of *ALK* fusions gradually increased, with significantly higher frequencies in mixed-IMA (20.0% vs. undetected, p=0.05) and pure-IMA (26.9% vs. undetected, p=0.01) when compared with non-IMA (**Figure S2B**). The *ALK* fusion was slightly more frequent in pure-IMA than mixed-IMA, with no statistically significant difference (26.9% vs. 20.0%, p=0.74) (**Figure 2B**). On the other hand, the frequency of *KRAS* mutation in pure-IMA was higher than that in non-IMA (23.1% vs. 12.0%) and mixed-IMA ad less *KRAS* mutation than non-IMA (4.0% vs. 12.0%), although none of these results were statistically significant (pure-IMA vs. non-IMA, p=0.47; mixed-IMA vs. non-IMA, p=0.36) (**Figure 2C** and **Figure S2B**). Besides, mixed-IMA tended to have more *ERBB2* 20Ins than pure-IMA (11.5% vs. 4.0%, p=0.35). *NRG1* fusion and *RET* fusion were individually observed in one patient from each of the two IMA cohorts of (**Figure S2B**).

### Different Profile of Tumor Suppressor Genes Between IMA and Non-IMA

Next, we investigated the mutational profile of tumor suppressors in patients with IMA and non-IMA. *TP53* (37.3% vs. 56.0%, p=0.14) showed a lower frequency while *RB1* (9.8% vs. 0%, p=0.17) showed a higher frequency in IMA than in non-IMA (**Figure 1**). In addition, the mutation frequency of other tumor suppressors, including *NF1*, *APC*, *PTEN*, and *STK11*, was higher in IMA than in non-IMA, albeit no significant difference (27.5% vs 12.0%, p=0.15). Interestingly, the frequency of co-mutations in these tumor suppressors was also higher in IMA than in non-IMA (19.6% vs 4.0%, p=0.09). In a specific subgroup of patients who harbored tumor suppressor mutations, the frequency of co-mutations in these suppressor genes was significantly higher in IMA patients than in non-IMA patients (40.0% vs 6.3%, p=0.003) (**Figure 1**). Similar to the results of *EGFR* mutations, as mucinous content increased, the frequency of *TP53* mutations gradually decreased (**Figure 1**). *TP53* mutations in pure-IMA were significantly more frequent than in non-IMA (23.1% vs. 56.0%, p=0.02) or mixed-IMA (23.1% vs. 52.0%, p=0.04), whereas no statistical difference was observed between non-IMA and mixed-IMA (p=1.0) (**Figure 2D**).

### No Difference in the Number of Somatic Mutations Between IMA and Non-IMA

**Figure 3:**
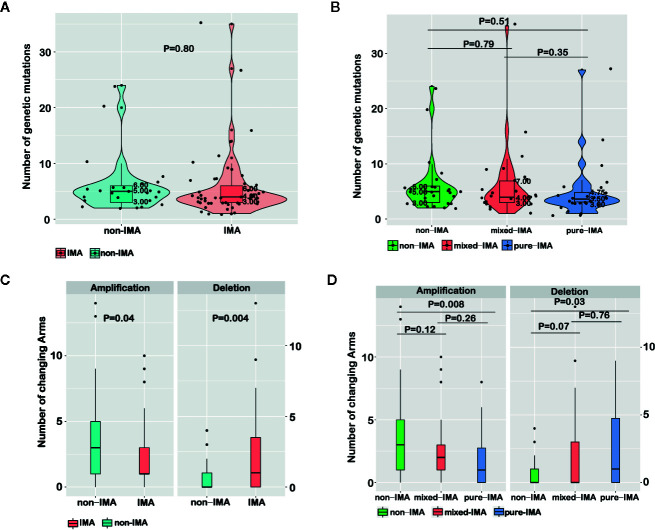
Comparing molecular features among samples with different mucinous contents. The number of genetic mutations for invasive mucinous adenocarcinoma (IMA) vs. non-IMA patients **(A)** or for patients that were divided based on the mucinous content **(B)** was shown in violin plots. The number of chromosome arm-level somatic CNVs (SCNVs) for IMA vs. non-IMA patients **(C)** or for patients that were divided based on the mucinous content **(D)** was shown in bar graphs.

We then compared the number of somatic mutations between non-IMA and IMA patients, including missenses, insertions/deletions (indels), fusions, and splice-site mutations. No significant difference in the number of somatic mutations was observed between IMA and non-IMA (p=0.8), and the median number was four and five in IMA and non-IMA, respectively (**Figure 3A**). Similarly, there was no significant difference in the number of mutations among non-IMA, mixed-IMA, and pure-IMA (**Figure 3B**).

### The Different Pattern of Chromosome Arm-Level SCNVs in Non-IMA and IMA

The chromosome arm-level SCNVs in non-IMA and IMA were analyzed. In IMA, the number of amplifications (p=0.04) was significantly lower while the number of deletions (p=0.004) was significantly higher than in non-IMA (**Figure 3C**). In detail, the frequencies of 5p (OR=0.34, 95% CI, 0.11–1.04; P=0.04), 6q (OR=0.11, 95% CI, 0.00–1.18; P=0.04), 8q (OR=0.21, 95% CI, 0.05–0.74; P=0.01), 16p (OR=0.14, 95% CI, 0.02–0.65; P<0.01), 16q (OR=0.17, 95% CI, 0.01–1.13; P=0.04), and 20q (OR=0.21; 95% CI, 0.06–0.68; P<0.01) amplifications were significantly lower in IMA than in non-IMA (**Table S1**). The frequency of 18q deletion in IMA was higher than that in non-IMA (OR=Inf; 95% CI, 0.9–Inf; P=0.05). The frequencies of 12p (OR=Inf; 95% CI, 0.74–Inf; P=0.09) and 18p (OR=6.48; 95% CI, 0.84–295.3; P=0.09) deletions in IMA were higher than that in non-IMA (**Table S1**). In addition, the arm-level SCNVs in patients with different proportions of the mucinous component were also analyzed. As the proportion of mucinous component increased, the frequency of amplification gradually decreased while the frequency of deletions gradually increased. There were also trends in arm-level SCNV differences when comparing non-IMA and mixed-IMA (amplification, p=0.12; deletion, p=0.07). Significant differences in SCNVs were observed between non-IMA and pure-IMA (amplification, p=0.008; deletion, p=0.03), whereas no significant difference was found between mixed-IMA and pure-IMA (p>0.05) (**Figure 3D**).

### Comparison of Mutation Profile Between East-Asian and Western IMA Patients

**Figure 4:**
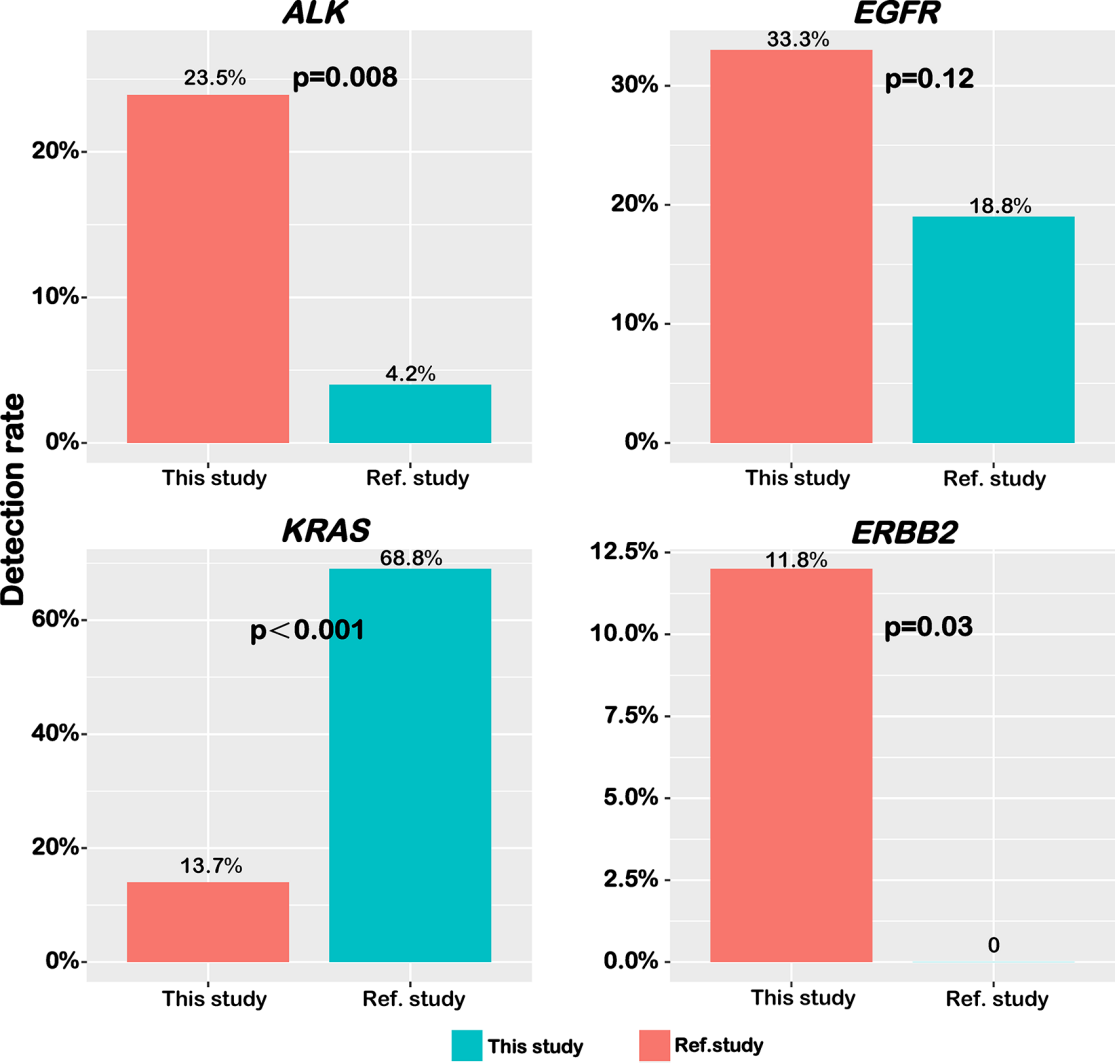
Distinct frequency of common driver genes between Chinese and Caucasian patients. The frequency of *ALK* fusion, *EGFR* mutation, *KRAS* mutation, and *ERBB2* mutation was shown in bar graphs.

Herein, a study conducting on Caucasians reported by Righi *et al.* (20) was selected as the reference study, in which 49 out of 50 genes used in their panel were also analyzed in our sequencing panel. In addition, patients in this reference study were mainly Western patients who were also divided into pure-IMA and mixed-IMA subgroups. Several driver genes that we analyzed above were then compared between the two patient cohorts. The frequency of *ALK* fusions (23.5% vs. 4.2%, p=0.008) and *ERBB2* mutations (11.8% vs. undetected, p=0.03) was significantly higher in our cohort while the frequency of *EGFR* mutations was comparable between the two cohorts (33.3% vs. 18.8%, P=0.12) (**Figure 4**). The frequency of *KRAS* mutations was significantly lower in our cohort (13.7% vs. 68.8%, p<0.001) (**Figure 4**). Furthermore, we compared the gene mutation profile between our cohort with the reference study in terms of the mucinous content. As shown in **Figure S3**, for mixed-IMA patients, there was a trend of higher, but not statistically significant frequency of *ALK* rearrangement (P=0.38) and *EGFR* mutation (P=0.33) in our study. *ERBB2* mutation also had the trend to be more frequent in our patients (P=0.06). However, there was almost no difference in the frequency of *TP53* and *APC* mutations (**Figure S3**). For pure-IMA patients, the frequency of *ALK* rearrangement was significantly higher in our study than in the reference study (26.9% vs. 3.3%, P=0.019), while the frequency of *EGFR* mutation was similar between the two studies (26.9% vs. 20.0%, p=0.52). Besides, there was almost no difference in mutation frequencies for *ERBB2* and *FLT3*. Mutations in *TP53* (23.1% vs. 76.5%, p=0.16) and *APC* (3.8% vs. 25.0%, p=0.11) were less in our cases, although no significant difference was observed. The frequency of *KRAS* mutations in both mixed-IMA and pure-IMA was significantly lower in our study than that in the reference study (p<0.001) (**Figure S3**).

### Individual Somatic Gene and Signaling Pathways Alterations Associated With Recurrence in Surgically Resected Patients With IMA

**Figure 5:**
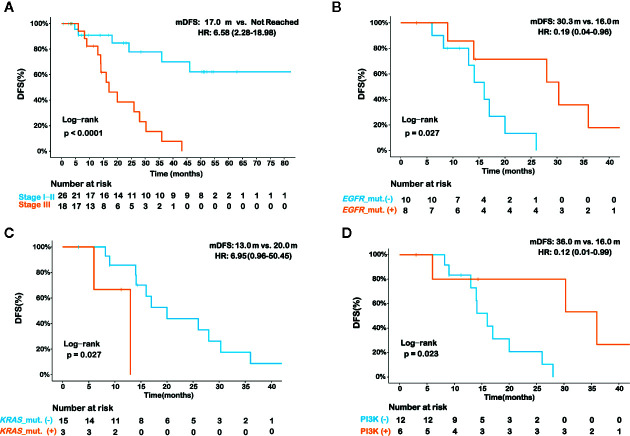
Prognostic analysis for various histopathologic and molecular features in invasive mucinous adenocarcinoma (IMA) patients. Kaplan-Meier curve of disease-free survival (DFS) in IMA patients in strata of different disease stages **(A)**. Kaplan-Meier curve of DFS in stage III IMA patients in strata of *EGFR* mutation status **(B)**, *KRAS* mutation status **(C)**, and PI3K signaling pathway alteration status **(D)**. The log-rank test was used to analyze the DFS for all of the survival analyses.

We then aimed to investigate whether the molecular profile in IMA patients affected their prognosis. We compared the postoperative disease-free survival (DFS) between patients with stage I/II tumors and stage III tumors. As shown in **Figure 5A**, the median DFS (mDFS) of patients with stage III disease was significantly worse than that of stage I/II (mDFS=17.0 months vs. Not reached; HR=6.58; 95% CI, 2.28–18.98; p<0.001). Due to the higher maturity of DFS in stage III patients, we further analyzed the correlations between molecular features and DFS in these patients. Survival data of 18 patients were available among the 21 stage III patients with IMA, and 14 of whom have relapsed after surgery. Our results showed patients with *EGFR* mutations had prolonged DFS than wildtype patients (**Figure 5B**). *EGFR-*mutated patients had an mDFS of 30.3 months, compared with an mDFS of 16.0 months in wildtype patients (HR=0.19; 95% CI, 0.04–0.96; P=0.027; **Figure 5B**). In contrast, patients with *KRAS* mutations showed shorter mDFS than those without *KRAS* mutations (mDFS=13.0 vs. 20.0 months; HR=6.95; 95% CI, 0.96–50.45; p=0.027) (**Figure 5C**). Previous studies reported that *TP53* mutations may lead to a worse prognosis in TKI-treated NSCLC patients; however, we did not observe significant differences in mDFS between patients with *TP53* mutations and those with wildtype *TP53* (mDFS=16.0 vs. 20.0 months; HR=1.18; 95% CI, 0.39–3.57; p=0.771) (**Figure S4A**). Moreover, in *EGFR*-mutated patients, no significant difference of mDFS was found in cases who had concomitant *TP53* mutations and those without *TP53* co-mutations (mDFS=30.3 vs. 28.0 months; p=0.810) (**Figure S4B**). As for other known oncogene drivers, such as *KRAS*, *ERBB2*, *RET*, *NRG1*, and *ALK*, there were only a limited number of patients harboring these oncogenic mutations or having available clinical results, thus the clinical impact of concomitant *TP53* mutations was still inconclusive in our patient cohort. Furthermore, the results of pathway analysis showed patients with genetic alterations in the PI3K pathway showed improved DFS (mDFS=36.0 vs. 16.0 months; HR=0.12; 95% CI, 0.02–0.99; P=0.023; **Table S2** and **Figure 5D**).

Lastly, we checked if some histopathologic features, such as intravascular tumor thrombus, perineural invasion, differentiation grading, and pleural invasion, were associated with prognosis in stage III IMA patients. By univariate analysis, we found that only the differentiation grading was significantly associated with DFS (**Table S3**). Specifically, IMA patients with moderately differentiated tumors had better DFS than those with poorly differentiated tumors (mDFS 28.0 vs. 14.1 mouths, p=0.037). In addition, we included both the differentiation grading and clinically-important molecular features that were identified in our study (**Figures 5B–D**) to perform a multivariate analysis. Intriguingly, DFS in these IMA patients remained to be significantly associated with *KRAS* mutation (p=0.016), PI3K pathway alteration (p=0.043), and differentiation grading (p=0.030), but not *EGFR* mutations (p=0.391) (**Table 2**), which might be due to the impact of *EGFR* mutations on lung adenocarcinomas differentiation, as previously described (31).

**Table 2:** Impact of genetic factors and differentiation grading on disease-free survival (DFS) in stage III patients with disease-free survival (IMA).

Variables	Univariate analysis		Multivariate analysis	
	HR (95% CI)	P_value	HR (95% CI)	P_value
*EGFR* (with vs. without)	0.19 (0.04–0.96)	0.027	2.49 (0.31–20.02)	0.391
*KRAS* (with vs. without)	6.95 (0.96–50.45)	0.027	85.15 (2.27–3,200.87)	0.016
PI3K pathway (with vs. without)	0.12 (0.01–0.99)	0.023	0.05 (0–0.91)	0.043
Differentiation grading (Poor vs. moderate)	3.75 (1.00–14.10)	0.037	17.14 (1.32–221.76)	0.030

## Discussion

In our study, the most commonly mutated driver gene in Chinese IMA patients was *EGFR*. The frequency of *EGFR* mutations was significantly lower while the frequency of *ALK* fusion was significantly higher in IMA compared to non-IMA, which was consistent with the previous studies (14–19, 32). The comparison of mixed-IMA and pure-IMA showed that there was no statistical difference in the frequency of *EGFR* or *ALK* variations, suggesting that these gene-level changes might happen in the entire tumor when lung adenocarcinoma harbors mucinous components. Interestingly, we observed a reverse trend in the frequency of *EGFR* and *ALK* alterations among patients with different proportions of the mucinous component, ranging from non-IMA, mixed-IMA, to pure-IMA. These results implied that adenocarcinoma with different proportion of mucinous component might be driven by distinct gene mutations.

In previous studies, *KRAS* mutation was more frequent in IMA, reaching as high as 63% (12, 17–19). In our study, the frequency of *KRAS* mutations was low in IMA (13.7%) and non-IMA (12.0%), which is likely due to the difference in *KRAS* mutation frequency between Caucasians and East-Asian populations (33). Of note, the frequency of *KRAS* mutations tended to be higher in pure-IMA compared to mixed-IMA (23.1% vs. 4.2%, p=0.1) in our East-Asian patient cohort, while almost no difference in *KRAS* mutation frequency was observed between pure-IMA and mixed-IMA in a study conducting on Caucasian patients (34). This suggested that, compared to Caucasians, there may be differences in the frequency of *KRAS* mutations in lung adenocarcinoma harbored different mucinous content in East-Asians. In a study performed on the Chinese population, reported by Chen et al., the frequency of *KRAS* mutations was 36%, which was higher than that of 13.7% in our study (19). As the mucinous content of IMA patients was not discussed in Chen *et al*.’s study, the detailed proportion of pure-IMA and mixed-IMA was unknown and, we speculated that the differences in *KRAS* mutation frequency may be partially due to the different proportion of pure-IMA patients.

In addition, multiple gene fusions, such as *NRG1*, *RET*, and *ALK*, were only detected in IMA patients in our cohort and the detecting rate (16/51, 31.4%) of gene fusions was significantly higher than the unselected lung adenocarcinoma patients (34). In addition, greater diversity in driver gene mutations in IMA compared to non-IMA was observed. For example, in a large cohort study of 10,966 Chinese NSCLC patients, the frequency of *NRG1* fusions was only 0.16% in these unselected lung cancer patients, with *CD74* being the most common fusion partner (35). In contrast, *NRG1* fusions occurred in 2 out of 51 (3.9%) IMA patients in our cohort, both of which harbored the *SLC3A2-NRG1* fusion that was previously reported by Jonna *et al.* (36) suggesting that *NRG1* fusions were highly enriched in the IMA subtype. For *NRG1* fusions, patients may benefit from the tyrosine kinase inhibitors (TKIs), e.g. afatinib (37). There are also several TKIs available for targeted therapy in advanced lung cancer patients with *RET* fusions, e.g. selpercatinib (38), pralsetinib (39), cabozantinib (40, 41), and vandetanib (42). In patients harboring *ALK* fusions, various ALK TKIs could be used in the clinical treatment of advanced NSCLC patients, such as crizotinib (43), alectinib (44–46), brigatinib (47, 48), ceritinib (49, 50), lorlatinib (51), ensartinib (52), and repotrectinib (53). Therefore, IMA patients may benefit from a broader option of TKIs compared to non-IMA patients.

Regarding tumor suppressors, *TP53* mutation was less in IMA, especially in pure-IMA. However, the mutation frequency of other tumor suppressors was higher in IMA than that in non-IMA. Moreover, the frequency of tumor suppressor co-mutations was significantly higher in IMA than non-IMA. This indicates that in IMA patients, once a tumor suppressor gene is involved in tumorigenesis, mutating additional tumor suppressor genes may have a synergistic effect. This may be a potential molecular mechanism underlying the poor prognosis of IMA patients, but studies with larger sample sizes are required for further verification.

In terms of the number of somatic mutations, no difference between IMA and non-IMA was observed. However, there were significant differences in SCNVs between IMA and non-IMA at the chromosome arm-level. There were more arm-level deletions and fewer arm-level amplifications in IMA compared to non-IMA. In addition, gradual decreasing and increasing trends of arm-level amplifications and deletions were observed from non-IMA to mixed-IMA to pure-IMA, respectively. These findings suggest that differences in molecular characteristics do exist between IMA and non-IMA and they may belong to different diseases.

Lastly, the survival-related analysis showed that the TNM stage was still the main factor affecting DFS after surgery, which is consistent with previous reports (54–56). In stage III IMA patients, cases who harbored *EGFR* mutations showed significantly prolonged DFS. According to previous studies, the prognostic impact of *EGFR* mutation status in patients with NSCLC remains controversial. Several articles have reported that the presence of *EGFR* mutations is a favorable prognostic factor in patients with surgically resected NSCLC (56–60). However, some other studies did not show a significant difference in survival between patients with *EGFR*-mutated tumors and those with wild-type after surgical resection (55, 61–64). In our study, we found that *EGFR* mutation is associated with improved DFS in stage III IMA patients. Similarly, patients with abnormalities in the PI3K signaling pathway displayed improved DFS. A previous study had suggested that lung adenocarcinoma patients with positive PI3K expression had a favorable survival, although it failed to be an independent prognostic predictor (65). In addition, Shan *et al*. found that phosphorylated AKT expression in the PI3K signaling pathway was a significant independent favorable prognostic factor in stage I to IIIA NSCLC (66). These findings suggest that the PI3K signaling pathway may be a potential favorable prognostic factor in patients with NSCLC. Conversely, patients with *KRAS* mutations have a poorer prognosis than those without *KRAS* mutations, which is consistent with previous studies conducted on unselected lung adenocarcinoma (54, 62, 67, 68). This suggests that *KRAS*-activating mutation is still an unfavorable prognostic factor in particular cases with IMA. As we did not have a large enough sample size, studies with more samples are required to further verify the prognostic values of abnormalities in *EGFR*, *KRAS*, and the PI3K pathway in IMA patients.

In summary, firstly, our study confirmed that the pattern of driver gene mutations between IMA and non-IMA was different, and IMA had greater diversity in mutations of driver genes. In addition, significant differences in the mutation frequency of some driver genes were observed between East-Asian and Caucasian populations, i.e. *ALK*, *KRAS*, and *ERBB2*. Secondly, we found that there were differences in the frequency of *EGFR*, *ALK*, *KRAS*, *TP53*, and arm-level SCNVs in tumors with different mucinous content. *EGFR* and *ALK* showed gradual decreasing and increasing trends from non-IMA to mixed-IMA to pure-IMA, respectively. *KRAS* mutation was more frequent in pure-IMA than in mixed-IMA. Compared to mixed-IMA and non-IMA, mutations in *TP53* were significantly enriched in pure-IMA. With regards to arm-level SCNVs, fewer amplifications but more deletions in IMA compared to non-IMA were observed, and these alterations also showed gradual decreasing and increasing trends from non-IMA to mixed-IMA to pure-IMA, respectively. Thirdly, co-mutations in tumor suppressors were more common in IMA compared to non-IMA, suggesting that this may be a potential molecular mechanism for poorer prognosis in IMA. Lastly, in stage III IMA patients, mutations in *EGFR* and the PI3K signaling pathway were associated with favorable postoperative DFS while patients with *KRAS* mutations or poorly differentiated tumors showed a shorter DFS. In conclusion, this study enhances our understanding of the genomic characteristics of IMA patients in an East-Asian population and helps us have a more comprehensive insight into this distinct histological subtype of lung adenocarcinoma.

## Data Availability Statement

The data presented in the study are deposited in the Genome Sequence Archive for Human (GSA-Human) repository (https://bigd.big.ac.cn/gsa-human/), accession number (HRA000507).

## Ethics Statement

The studies involving human participants were reviewed and approved by Medical Ethics Committee of Zhejiang Cancer Hospital. The patients/participants provided their written informed consent to participate in this study. Written informed consent was obtained from the individual(s) for the publication of any potentially identifiable images or data included in this article.

## Author Contributions

Study concept and design: YJ, LC, and JW. Analysis and interpretation of data: JY, YX, FW, YS, and QX. Clinical information collection: LC, JW, JZ, JL, CP, XH, GX. MX and XX. Pathological evaluation: LZ and JJ. All authors contributed to the article and approved the submitted version.

## Funding

This study was supported by the grants from the National Natural Science Foundation of China Grants (No. 81802995 and No. 81702851), and the Medical Science and Technology Project of Zhejiang Province (2018KY024).

## Conflict of Interest

Authors JY, YX, FW, YS and QX were employed by Nanjing Geneseeq Technology Inc. 

The remaining authors declare that the research was conducted in the absence of any commercial or financial relationships that could be construed as a potential conflict of interest.

## Supplementary Material

Supplementary Figure 1 The landscape of somatic mutations in all 76 patients in our cohort. The oncoprint only shows the mutated genes occurring in at least 3 patients of all the patients.

Supplementary Figure 2 Pie chart showing the known driver mutations detected in IMA and non-IMA **(A)** or in mixed-IMA and pure-IMA **(B)** patients.

Supplementary Figure 3 Comparison of gene mutations in pure-IMA or mixed-IMA between Chinese and Caucasian patients. *EGFR* and the other genes with p-value < 0.2 in any group of mixed-IMA or pure-IMA were shown here.

Supplementary Figure 4 Prognostic analysis of TP53 mutations in IMA patients. Kaplan-Meier curve of DFS in strata of TP53 mutation status in all stage III patients **(A)**, and concomitant TP53 mutation status in EGFR-mutated stage III patients **(B)**. The log-rank test was used to analyze the DFS for the survival analyses.
