# Supplementary figures and images for: Genomic Profiling and Prognostic Value Analysis of Genetic Alterations in Chinese Resected Lung Cancer With Invasive Mucinous Adenocarcinoma
Source: Front Oncol. 2021 Jan 11;10:603671. doi: 10.3389/fonc.2020.603671 (PMC7829865; doi:10.3389/fonc.2020.603671)

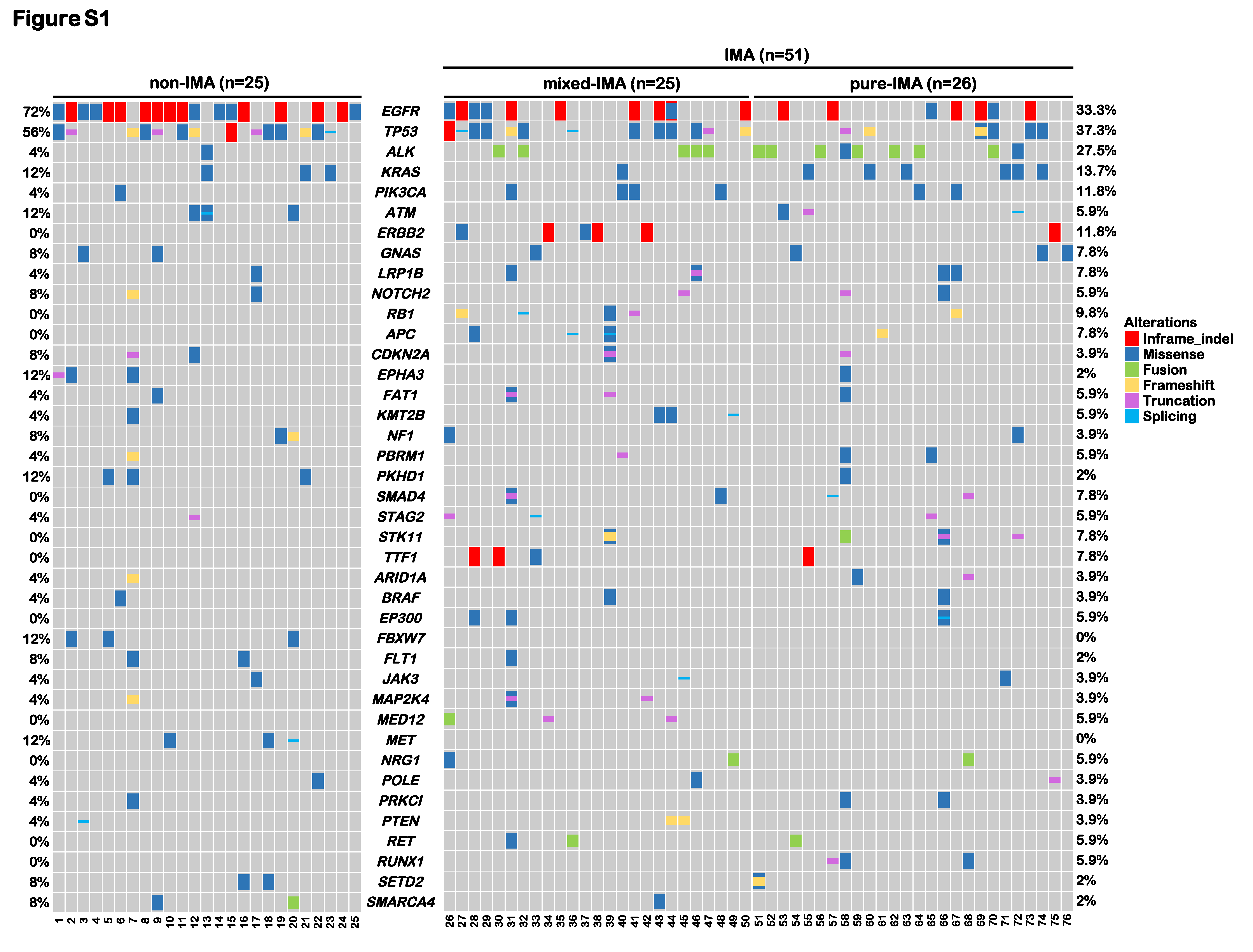

Supplement: Supplementary Figure 1 — The landscape of somatic mutations in all 76 patients in our cohort. The oncoprint only shows the mutated genes occurring in at least 3 patients of all the patients. [file Image_1.tiff]

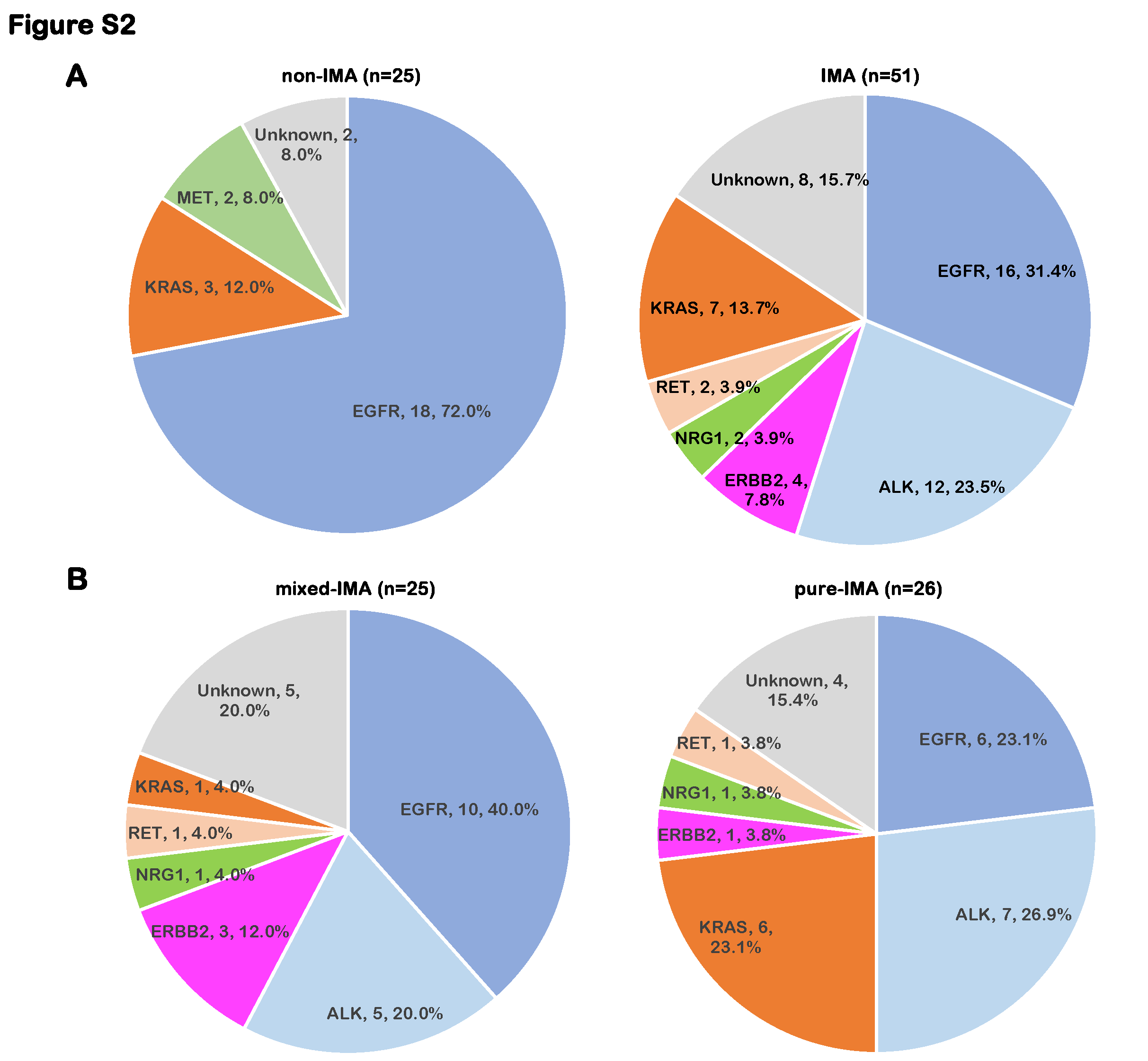

Supplement: Supplementary Figure 2 — Pie chart showing the known driver mutations detected in IMA and non-IMA (A) or in mixed-IMA and pure-IMA (B) patients. [file Image_2.tif]

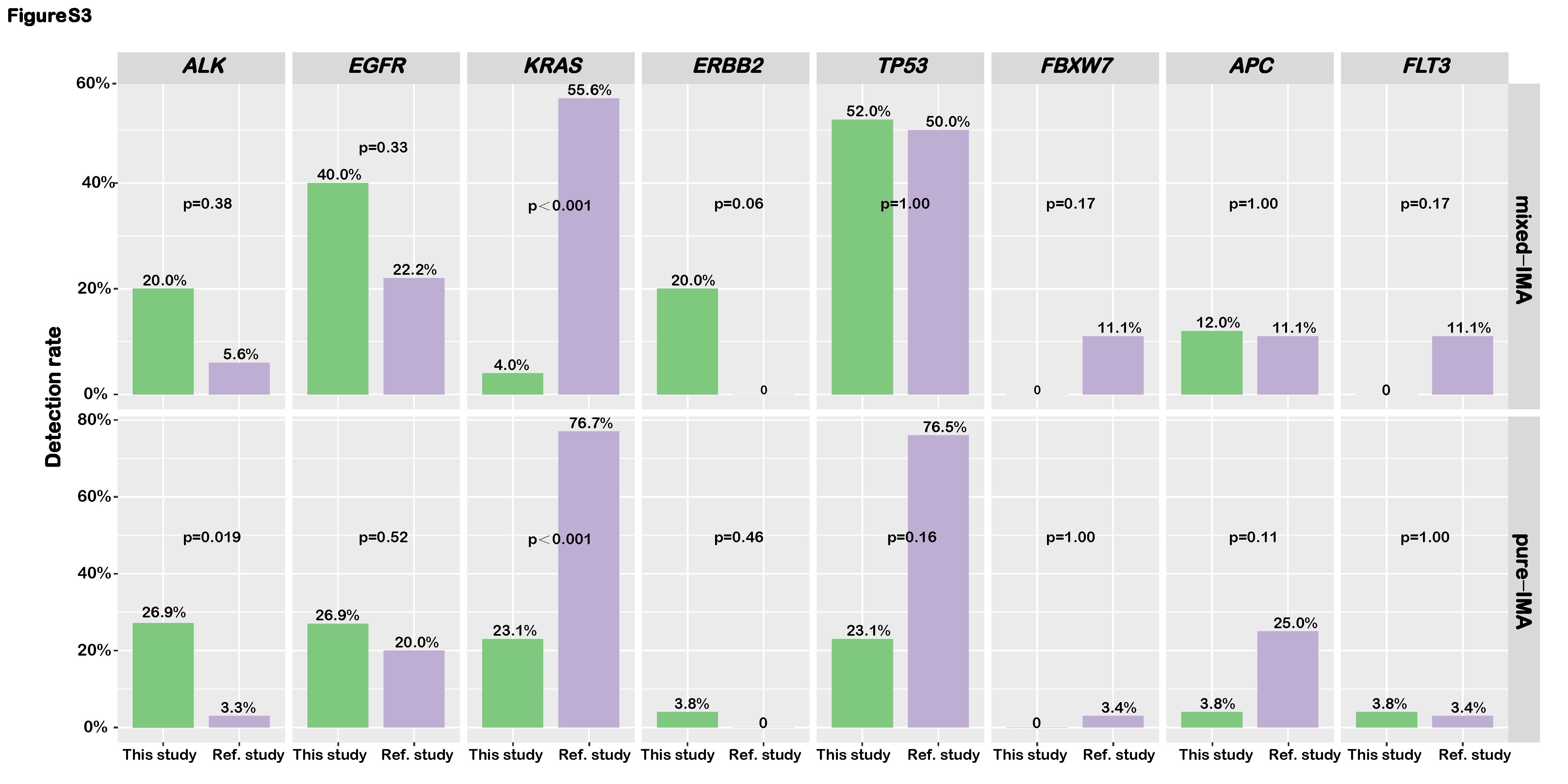

Supplement: Supplementary Figure 3 — Comparison of gene mutations in pure-IMA or mixed-IMA between Chinese and Caucasian patients. EGFR and the other genes with p-value < 0.2 in any group of mixed-IMA or pure-IMA were shown here. [file Image_3.tif]

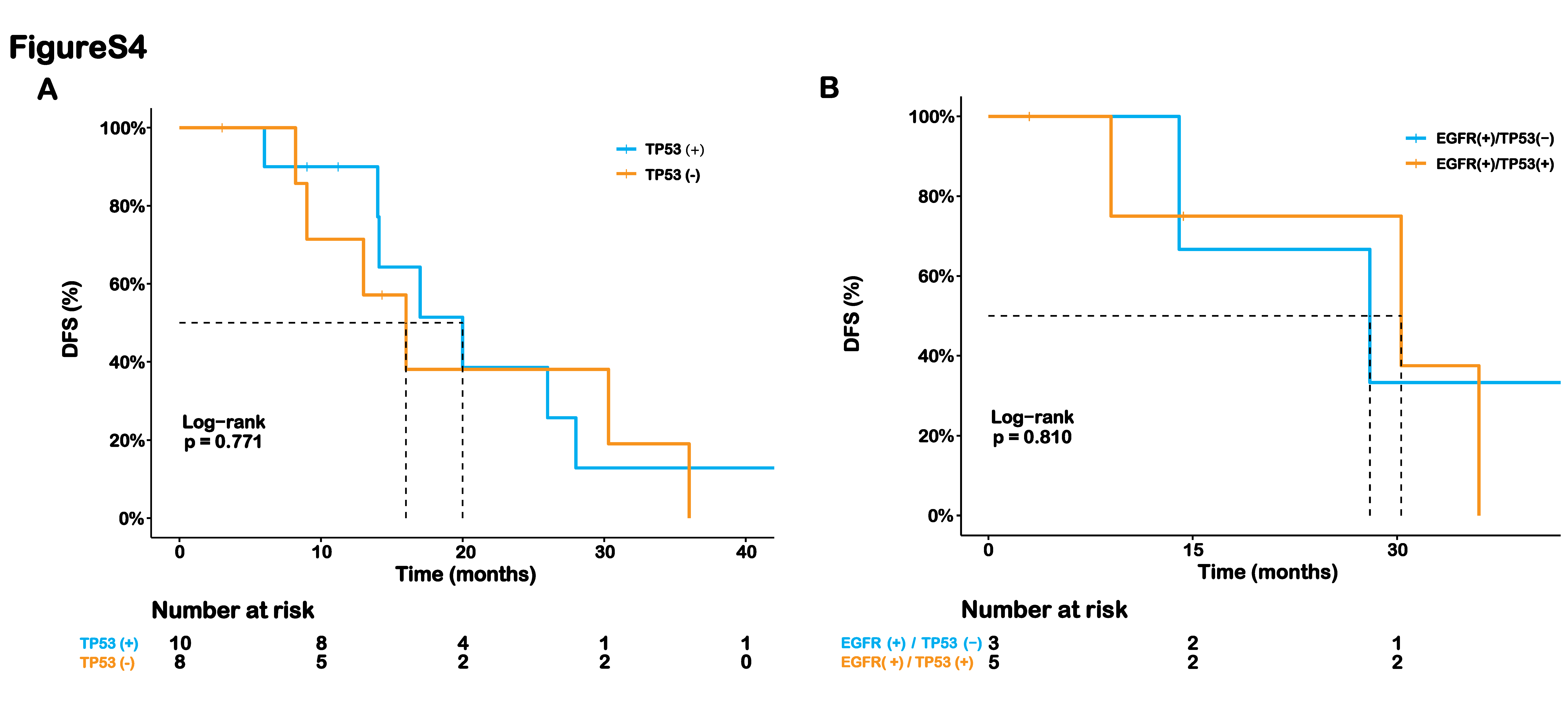

Supplement: Supplementary Figure 4 — Prognostic analysis of TP53 mutations in IMA patients. Kaplan-Meier curve of DFS in strata of TP53 mutation status in all stage III patients (A), and concomitant TP53 mutation status in EGFR-mutated stage III patients (B). The log-rank test was used to analyze the DFS for the survival analyses. [file Image_4.tif]
