# Supplementary material for: Genomic Profiling and Prognostic Value Analysis of Genetic Alterations in Chinese Resected Lung Cancer With Invasive Mucinous Adenocarcinoma
Source: Front Oncol. 2021 Jan 11;10:603671. doi: 10.3389/fonc.2020.603671 (PMC7829865; doi:10.3389/fonc.2020.603671)
Supplement: Supplementary file 5 [file Table_1.docx]

Table S1. Chromosome arm-level SCNAs in IMA patients compared to the Non-IMA patients (Fisher exact test, p-value＜0.1).

| **Arm level alterations** | **Arm position** | **OR (95% CI)** | **P-value** |
| --- | --- | --- | --- |
| Amplification | 5p | 0.34 (0.11-1.04) | 0.04 |
|  | 6q | 0.11 (0-1.18) | 0.04 |
|  | 8q | 0.21 (0.05-0.74) | 0.01 |
|  | 16p | 0.14 (0.02-0.65) | <0.01 |
|  | 16q | 0.17 (0.01-1.13) | 0.04 |
|  | 20q | 0.21 (0.06-0.68) | <0.01 |
| Deletion | 12p | Inf (0.74-Inf) | 0.09 |
|  | 18q | Inf (0.9-Inf) | 0.05 |
|  | 18p | 6.48 (0.84-295.3) | 0.09 |

OR: Odds ratio; Inf: Infinity.
