# Supplementary material for: Genomic Profiling and Prognostic Value Analysis of Genetic Alterations in Chinese Resected Lung Cancer With Invasive Mucinous Adenocarcinoma
Source: Front Oncol. 2021 Jan 11;10:603671. doi: 10.3389/fonc.2020.603671 (PMC7829865; doi:10.3389/fonc.2020.603671)
Supplement: Supplementary file 6 [file Table_2.docx]

Table S2. Univariate Cox regression analysis of DFS with signaling pathways in stage III patients with IMA.

| **Pathway** | **No. of patients** | **HR (95% CI)** | **Logrank_p value** |
| --- | --- | --- | --- |
| Cell_cycle | 5 | 0.548 (0.144~2.087) | 0.372 |
| HIPPO | 1 | 0 (0~Inf) | 0.478 |
| NOTCH | 3 | 1.239 (0.262~5.859) | 0.786 |
| PI3K | 6 | 0.120 (0.015~0.991) | 0.023 |
| TGF_β | 2 | 0.505 (0.064~3.973) | 0.508 |
| TP53 | 10 | 1.965 (0.63~6.121) | 0.236 |
| Wnt | 3 | 0.321 (0.068~1.52) | 0.135 |
| RTK_RAS | 17 | 0.700 (0.085~5.768) | 0.739 |

Inf: Infinity.
