# Supplementary material for: Genomic Profiling and Prognostic Value Analysis of Genetic Alterations in Chinese Resected Lung Cancer With Invasive Mucinous Adenocarcinoma
Source: Front Oncol. 2021 Jan 11;10:603671. doi: 10.3389/fonc.2020.603671 (PMC7829865; doi:10.3389/fonc.2020.603671)
Supplement: Supplementary file 7 [file Table_3.docx]

Table S3. Univariate Cox regression analysis of DFS with histopathologic features in stage III patients with IMA.

| **Variables** | **HR (95% CI)** | **Logrank_p value** |
| --- | --- | --- |
| Intravascular tumor thrombus  (Yes vs. No) | 0.97 (0.31~3.09) | 0.965 |
| Perineural invasion  (Yes vs. No) | 0.38 (0.05~3.14) | 0.353 |
| Differentiation grading  (Poor vs. Moderate) | 3.75 (1.00~14.10) | 0.037 |
| Pleural invasion  (Yes vs. No) | 1.04 (0.34~3.16) | 0.951 |
